# Metastatic renal cell carcinoma complicated by right atrial thrombus

**DOI:** 10.1002/ccr3.4028

**Published:** 2021-03-11

**Authors:** Hafiz Muhammad Abrar Jeelani, Muhammad Mubbashir Sheikh, Adeel Riaz, Nikita Jain, Nayha Tahir, Hamid Ehsan, Lalitha Vemireddy

**Affiliations:** ^1^ Rosalind Franklin University/Northwestern Medicine McHenry Hospital McHenry IL USA; ^2^ University of Pittsburgh Medical Center Pittsburgh PA USA; ^3^ Punjab Medical College Faisalabad Pakistan; ^4^ MedStar Union Memorial Hospital Baltimore MD USA

**Keywords:** cardiovascular disorders, nephrology, oncology

## Abstract

Metastasis of renal cell carcinoma to the heart is a rare event. Herein we present a case of renal cell carcinoma presenting with progressive fatigue, abdominal pain, and weight loss. Imaging studies revealed complex renal mass with extension to right atrium and histopathology confirmed the metastatic renal cell carcinoma.

## Graphical abstract

Patients with renal cell carcinoma not always present with classic clinical features. Metastatic involvement of the right atrium is an exceedingly rare event and carries a poor prognosis. Our case highlights the importance of diagnosing renal cell carcinoma at early stages to prevent future morbidities.

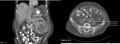

## 1 INTRODUCTION

Kidney cancer constitutes about 3% of all malignancies in the adult population.^1^ It is the 7th most common cancer in men and the 10th most common cancer in women.^2^ More than 400 000 new cases of renal cell carcinoma (RCC) were reported globally in 2018.^3^ Each year, roughly 63 000 new RCC cases are diagnosed with 14 000 deaths in the United States (US).^4^ It is twice as common in men as compared to women.^5^ The classic triad of palpable flank pain, flank mass, and hematuria for RCC are usually seen in advanced cases.^6^ RCC is associated with coagulopathy and development of inferior vena cava (IVC) thrombus in 4%‐10% of cases, with approximately only 1% extending into the right atrium.^1^ Metastasis is common to the lungs, liver, lymph nodes, brain, bones, and a significant predictor of prognosis in RCC.^7^ With the increasing use of imaging, more than 70% of all RCC being diagnosed as an incidental finding on imaging studies.^7^ The most common histological type of RCC is clear cell cancer (70%‐80%), followed by papillary renal cell cancer (10%‐20%).^7^ It is more common in patients with hypertension, hyperlipidemia, smoking, end‐stage renal disease, acquired cystic renal disease, kidney transplantation, and tuberous sclerosis syndrome.^8^ More than 30% of patients with RCC found to have metastatic disease at the time of diagnosis.^9^ The five‐year survival with localized RCC is 92.1%, with the regional disease is 65.4%, and with metastatic RCC is only 11.8%.^10^ A combination of cytoreductive nephrectomy with systemic targeted therapies is currently being used for metastatic RCC due to tumors' highly immunogenic nature.^11^ Radical surgical resection remains the definitive curative and palliative treatment in patients with IVC thrombus propagating to the right atrium without significant systemic metastases.^12^ However, IVC thrombectomy is associated with significant morbidity and mortality and requires careful patient selection with a multidisciplinary care team approach for better outcomes.^12^

## 2 CASE PRESENTATION

A 66‐year‐old woman with a past medical history of hypertension, hyperlipidemia, hypothyroidism initially presented to the outpatient clinic with complaints of fatigue, generalized weakness, and unintentional weight loss of 10 pounds over three months. She was found to have anemia, and iron with vitamin D supplements was started. The patient was noted to have worsening of her symptoms with nausea, abdominal/flank pain. She also reported insomnia, bilateral pedal edema, loss of appetite, abdominal bloating, and abdominal fullness despite small portions of food. Colonoscopy was done, which was unremarkable.

**FIGURE 1:**
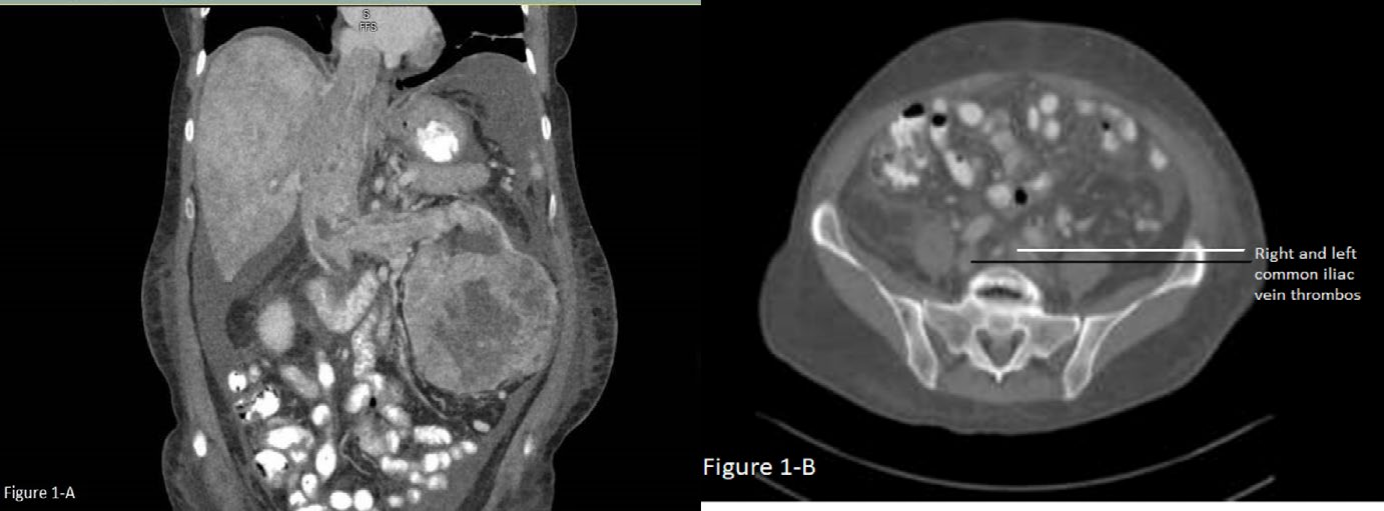
Computed tomography results of abdomen and pelvis showing left renal mass. 1A: Left kidney mass measuring up to 11.6 cm into 10.7 cm in trans‐axial dimension and up to 10.2 cm craniocaudal, with tumor thrombus involving left renal vein and extending cranially via inferior vena cava (Coronal View) 1B: Caudal extension of tumor thrombus with bilateral common iliac veins involvement (Cross‐sectional view)

A computed tomography (CT) scan of the abdomen and pelvis with oral and intravenous (IV) contrast showed large complex mixed attenuation partly solid and cystic mass, occupying the majority of mid to lower half of the left kidney measuring up to 11.6 cm into 10.7 cm in trans‐axial dimension and up to 10.2 cm craniocaudal as shown in Figure 1A. There was a contiguous extension of this mass‐like structure into the renal hilum and across the left renal vein with a marked expansion of the left renal vein to approximately 3.5 cm in diameter with continued extension into the massively dilated inferior vena cava measuring up to 4.7 cm in diameter. Within the inferior vena cava, there was a filling defect tracking superiorly to the cavo‐atrial junction, partially protruding into the right atrium and showing extension into the right hepatic vein. There was a hypodensity in the distal inferior vena cava progress to bilateral common iliac veins and external iliac veins (Figure 1B). A significant enlargement of the azygous vein, likely representing a collateral blood flow, was also noted. However, there was also a patent periphery to the inferior vena cava, probably representing venous flow around the tumor tissue itself. Associated hepatic congestion, venous collateral formation, ascites, and edema were also found on imaging (Figure 1A).

Physical examination was remarkable for tachycardia to 112 beats per minute, relative hypotension with a blood pressure of 94/52 mm of Hg, abdominal distension, left‐sided abdominal pain, flank tenderness with fullness, and a palpable mass. Laboratory parameters were within normal range.

**FIGURE 2:**
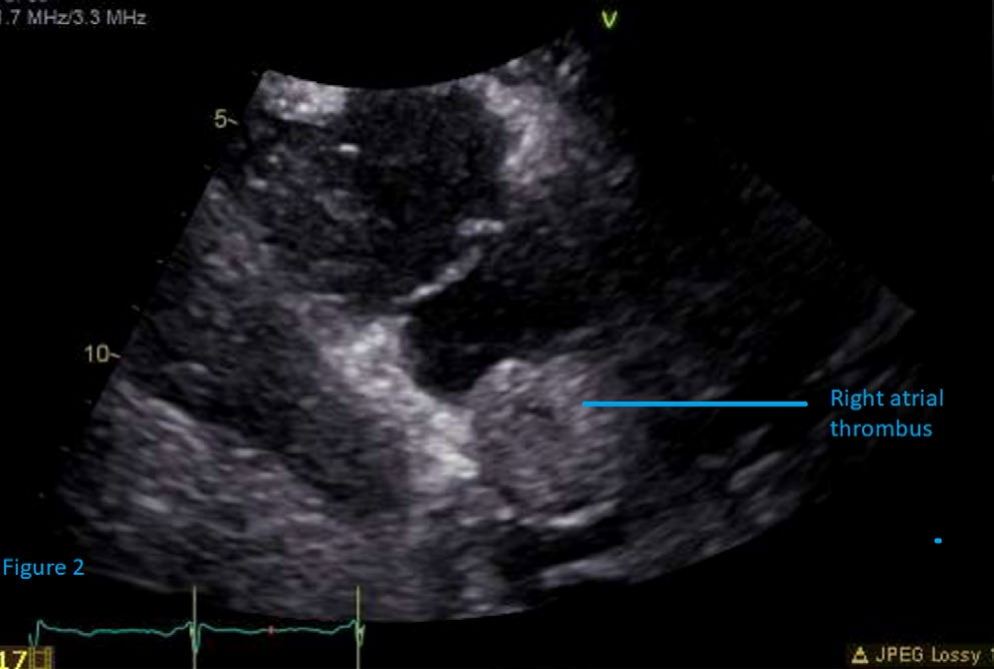
Echocardiogram showing right atrial thrombus (Blue line)

**FIGURE 3:**
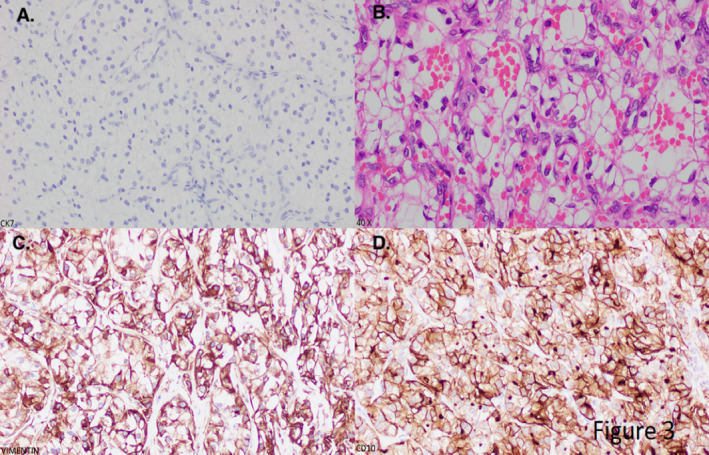
Histopathology examination left renal mass. Positive immunohistochemical for CK7 (panel A), clear cells (panel B), positive immunohistochemical for vimentin, and positive immunohistochemical stain for CD‐10 (panel D)

The patient was started on intravenous heparin infusion due to a high risk of pulmonary embolism and transferred to the intensive care unit (ICU) for close monitoring. The oncology, interventional radiology (IR), cardiology, and surgery consults were requested. An echocardiogram of the heart showed a left ventricular ejection fraction of 71%. The right atrial cavity was normal in size. A mass was protruding into the right atrium, measuring up to 2.8 cm into 3.1 cm extending into the right atrium from the inferior vena cava (Figure 2). Ultrasound‐guided paracentesis was done, and fluid was negative for malignant cells. Chest X‐ray (CXR) showed normal cardiac shadow with small bilateral pleural effusions. Image‐guided biopsy of the left renal mass was done, and histopathology showed renal cell carcinoma favoring clear cell type with strong affinity for cluster of differentiation‐10 (CD10), cytokeratin‐7 (CK7), and vimentin (Figure 3). The tumor was categorized as left‐sided renal cell carcinoma stage III (cT3cN0M0) by an oncologist. Treatment began with a long‐term therapeutic dose of lovenox and dual immunotherapy with ipilimumab and nivolumab. She was not a candidate for surgical and interventional radiology (IR)‐guided interventions due to hepatic vein thrombus and the extensive nature of thrombus.

## 3 DISCUSSION

Renal cell carcinoma is an aggressive tumor that classically presents with flank pain, abdominal mass, and hematuria only in advanced cases. According to the National Cancer Institute Surveillance, Epidemiology, and End Results (SEER) program, there will be an estimated 73 750 new RCC cases in the United States in 2020, constituting 4.1% of all newly diagnosed cancer cases.^10^ Data for 2021 are not available to date. RCC mostly presents as a localized or locally advanced mass, and about 30% of patients have disease metastasized to different sites at the time of diagnosis. The most common metastatic sites are the central nervous system, adrenals, lungs, liver, and intra‐abdominal structures. The heart is an uncommon and atypical site of metastasis for RCC with an incidence of 1.3%‐4.2%.^13^ The primary histopathological type of cardiac metastasis is the clear cell RCC. The primary mechanism of RCC metastasis to the heart is a direct extension of intravascular tumor growth into renal veins and inferior vena cava, thus seeding the heart tissue. RCC has a high propensity to invade local vasculature extending to renal veins and inferior vena as a solid column, with 1% of cases having extension up to the right atrium level. In our patient, the imaging studies showed that the left lower kidney mass had contiguous extension across the renal vein with continuous extension into the inferior vena cava tracking cranially to cavo‐atrial junction partially protruding into the right atrium. Our patient's unique feature was the extension of the tumor in the caudal direction inferior to the renal veins in the distal inferior vena cava, bilateral common iliac veins, and external iliac veins. No such extension has been demonstrated in the published data.

Management of RCC, either medically or surgically, depends on the stage of the disease. CT scan and magnetic resonance imaging (MRI) are preferred imaging modalities for staging the disease, with MRI being the gold standard.^14^ Echocardiography and CXR are used to locate and explore cardiac disease. In our case, the CT scan revealed findings, as mentioned above. Echocardiography showed a plump‐shaped mass protruding from the IVC in the right atrium, measuring 2.8 cm into 3.1 cm. Surgical resection of tumors with nephrectomy and removal of IVC thrombus is the standard of care but only in patients with expected good performance status as the procedure is associated with significant morbidity and mortality.^14^ Patients who are not surgical candidates, as our patient, due to extensive disease or contraindications to surgery, are managed with chemotherapy, hormonal, or radiation therapy; however, targeted therapy with agents such as ipilimumab, nivolumab sorafenib, sunitinib, temsirolimus, everolimus, and axitinib is the standard of care these days. Ipilimumab plus nivolumab showed superior efficacy over others.^15^ However, the long‐term prognosis is dismal in metastatic RCC with prognosis in months even with aggressively targeted immunotherapy.

## 4 CONCLUSIONS

Renal cell carcinoma is associated with widespread metastatic disease and a hyper‐coagulopathic state. It typically metastasizes to unpredictable sites with atypical symptoms and extension of thrombus into IVC and right atrium. The classic triad of flank pain, flank mass, and hematuria is usually seen in advanced disease stages. These days the majority of cases are diagnosed as incidental findings on imaging studies for other reasons. Therefore, it is highly essential to consider RCC in the differential when the patient presents with atypical symptoms without any workable diagnosis, as early stages of RCC are highly responsive to standard therapy with a good prognosis and long‐term survival.

## CONFLICT OF INTEREST

The authors report no conflict of interest.

## AUTHOR CONTRIBUTIONS

Hafiz Muhammad Abrar Jeelani: contributed to conception of idea, acquisition of data, manuscript writing, and final approval. Muhammad Mubbashir Sheikh: contributed to acquisition of data, critical revision, manuscript writing, and final approval. Adeel Riaz: contributed to critical revision, manuscript writing, and final approval. Hamid Ehsan, Lalith Vemireddy, Nayha Tahir, and Nikita Jain: contributed to manuscript writing and final approval.

## ETHICAL APPROVAL

This manuscript did not receive any financial support.

## Data Availability

The data will be made available on the request due to ethics and privacy.
